# Early-life predictors of resilience and related outcomes up to 66 years later in the 6-day sample of the 1947 Scottish mental survey

**DOI:** 10.1007/s00127-016-1189-4

**Published:** 2016-02-15

**Authors:** Mathew A. Harris, Caroline E. Brett, John M. Starr, Ian J. Deary, Andrew M. McIntosh

**Affiliations:** Centre for Cognitive Ageing and Cognitive Epidemiology, University of Edinburgh, 7 George Square, Edinburgh, EH8 9JZ UK; Alzheimer Scotland Dementia Research Centre, University of Edinburgh, Edinburgh, UK; Division of Psychiatry, University of Edinburgh, Edinburgh, UK

**Keywords:** Resilience, Personality, Early-life stress, Health and wellbeing, 6-day sample

## Abstract

**Purpose:**

Psychological resilience, the ability to manage and quickly recover from stress and trauma, is associated with a range of health and wellbeing outcomes. Resilience is known to relate to personality, self-esteem and positive affect, and may also depend upon childhood experience and stress. In this study, we investigated the role of early-life contributors to resilience and related factors in later life.

**Methods:**

We used data from the 6-day sample of the Scottish mental survey 1947, an initially representative sample of Scottish children born in 1936. They were assessed on a range of factors between the ages of 11 and 27 years, and resilience and other outcomes at 77 years.

**Results:**

Higher adolescent dependability unexpectedly predicted lower resilience in older-age, as did childhood illnesses, while a count of specific stressors experienced throughout early life significantly predicted higher later-life resilience. We also observed significant cross-sectional correlations between resilience and measures of physical health, mental health, wellbeing and loneliness. Some of the associations between early-life predictors and later-life outcomes were significantly mediated by resilience.

**Conclusions:**

Our results support the hypothesis that stress throughout early life may help to build resilience in later-life, and demonstrate the importance of resilience as a mediator of other influences on health and wellbeing in older age. We suggest that the mechanisms determining how early-life stress leads to higher resilience are worthy of further investigation, and that psychological resilience should be a focus of research and a target for therapeutic interventions aiming to improve older-age health and wellbeing.

## Introduction

Life can be stressful, but the long-term effects of stress depend on how it is processed. Adaptation to stress is often referred to as vulnerability or resilience, depending on whether risk of psychiatric illness is conferred or mitigated. Specifically, psychological resilience is the ability to positively adapt to adversity [1] and effectively manage the associated trauma [2], facilitating recovery from stressful events [3]. An increasing amount of research is focused on psychological resilience and its underlying mechanisms, due to its clear association with important health and wellbeing outcomes. Smith and colleagues [4], testing their Brief Resilience Scale in student samples, observed moderate negative correlations of −0.41 to −0.56 between resilience, and depression and anxiety. Others have shown similar associations between resilience and mental health incidence and severity in other populations [5–7]. While physical resilience is defined in terms of positive outcomes to health problems, higher psychological resilience (generally assessed using questionnaires) has also been associated with better physical health [6], including reduced symptom severity in a range of physical illnesses [4, 8]. As morbidity increases with ageing, psychological resilience may be particularly relevant in later life.

Resilience has been closely associated with personality traits. Campbell-Sills, Cohan and Stein [9] assessed the relations between scores on the Connor–Davidson Resilience Scale and the Big Five traits in a sample of 132 undergraduates. They observed a strong negative correlation between resilience and neuroticism (−0.65), and positive associations with extraversion (0.61) and conscientiousness (0.46). Nakaya, Oshio and Kaneko [10] produced very similar results using the Adolescent Resilience Scale. Further studies have demonstrated that individual differences in personality account for approximately 40 % of variance in resilience scores [11, 12]. Other factors positively associated with resilience include positive affect [3], self-esteem and optimism [13], secure attachment in early childhood [3], parental support in later childhood [14] and perceived social support in adulthood [15]. Friborg et al. [11] also suggested that individuals of higher intelligence should be able to apply their cognitive abilities to coping with adversity. However, they did not find evidence to support their hypothesis, and, to our knowledge, none have since assessed the relationship between resilience and intelligence.

Stress in childhood may also be associated with higher resilience in adulthood. Martin and Martin [16] argued that being able to cope with recent stressful events, maintaining a positive developmental outcome depends upon resources that develop in response to childhood adversity. Lyons and colleagues [17, 18] present evidence from longitudinal primate studies for ‘stress inoculation’, indicating that stress, if not too severe, promotes resilience. Shapero et al. [19] observed stress inoculation in humans; among 163 adolescents studied for 3 years, moderate stress during childhood (prior to age 12–13 years) reduced the risk of a depressive response to stress in adolescence (up until age 15–16 years). However, others propose that those exposed to higher levels of stress in early life can become less able to effectively manage subsequent stress, and that this ‘stress sensitisation’ is associated with a range of psychiatric disorders [20, 21]. Whether inoculation or sensitisation occurs in reaction to stress may be determined by individual differences in glucocorticoid responsivity or innate coping strategies, or by external factors such as support from others. Alternatively, it could be the severity of stress; Middlebrooks and Audage [22] argue that, while ‘positive stress’ is important for the development of coping skills, ‘toxic stress’ can be severely detrimental to development. They also suggest an intermediary category of ‘tolerable stress’, the result of which depends upon individual differences and environmental factors. As of yet, the effects of early-life stress on later resilience are poorly understood, as are the effects of early stress inoculation or sensitisation on later-life outcomes.

The present study investigated the contributions of various early-life factors, recorded between 11 and 27 years of age, to psychological resilience in later life, measured by questionnaire at age 77. We used data from the 6-Day Sample, an initially representative sample drawn from the 1947 Scottish mental survey of Scottish children born in 1936 [23–25]. We aimed to identify whether factors influencing development—IQ, personality, environmental circumstances and adversity in childhood, and stressors throughout early adulthood—predicted resilience up to 66 years later. We hypothesised that childhood dependability would be positively correlated with later resilience, in line with previous evidence of a positive relationship between similar personality traits and resilience. As more intelligent individuals should be able to apply their cognitive abilities to overcoming adversity, we also expected a positive correlation between childhood intelligence and later resilience. All other predictors—childhood illness, social deprivation, home and school moves, parents’ deaths or separation, and similar measures of deprivation and trauma throughout early-adulthood—were treated as stressors, expected to predict either higher resilience due to stress inoculation, or lower resilience due to sensitisation.

To confirm the relevance of resilience in older age, we also assessed its contemporaneous associations with physical health, mental health, subjective wellbeing, loneliness and cognitive ability. In line with previous findings, we expected higher resilience to be associated with better physical health, fewer mental illness symptoms, higher subjective wellbeing and lower loneliness. We also speculated that resilience may protect against cognitive decline, hypothesising that higher resilience would be associated with higher cognitive ability in older age, and relatively reduced cognitive decline from childhood to older age. Finally, we expected that some of the early-life variables predicting resilience would show direct associations with those related to resilience in older age, and that these relationships would be mediated by resilience.

## Methods

### Participants

Almost all children born in 1936 and present at schools in Scotland on 4th June 1947 completed the second Scottish mental survey (SMS1947) [26]. A sample of this cohort were selected for further study and confirmed to be representative of the population [27]. As participants were chosen based on their dates of birth being on 1 of 6 days throughout the year, this sample was referred to as the ‘6-Day Sample’. Participants were studied in further detail at school and through a series of almost annual home visits up until age 27 [23]. Of the original 1208 sample members, 634 were traced through the UK National Health Service Central Register as alive and resident in Scotland, England or Wales at the beginning of the follow-up study [28]. In response to invitations sent to these 634 people (and one other who had emigrated), 174 (91 female) completed an initial questionnaire, and 131 (72 female) subsequently completed a telephone interview. Participants were first tested in 1947 at the mean age of 10.9 (SD 0.3), and completed the questionnaire and telephone interview in 2013 at the mean ages of 76.7 (SD 0.4) and 77.1 (SD 0.3), respectively. Further details are included in Table 1. Those who completed the follow-up study had higher mean scores on the Moray House Test of intelligence and were rated by their teachers as more dependable than the population as a whole [25, 29].

**Table 1:** Descriptive statistics for participants in each stage of the study

	SMS1947		6-day sample		Followed up		Tel. interviewed	
	Mean	SD	Mean	SD	Mean	SD	Mean	SD
*N*	70,805		1208		171		129	
Sex (M/F)	35,809/34,996		590/618		82/89		59/70	
Age	10.9	0.29	10.9	0.28	76.7	0.40	77.1	0.34
Early life								
MHT score	36.9	15.8	37.4	15.8	48.1	11.6	49.2	11.4
SB IQ			102.6	20.1	115.7	19.7	118.7	19.1
Dependability			9.4	2.2	10.3	2.2	10.5	2.2
Childhood illnesses			0.39	0.68	0.47	0.75	0.45	0.76
Social class			3.37	0.97	3.11	1.02	3.06	1.00
Family size			3.76	2.28	3.16	1.77	3.02	1.64
Birth order			2.41	1.76	2.08	1.43	2.08	1.40
Home occupancy			1.70	0.76	1.42	0.67	1.39	0.62
Home and school moves			3.84	2.12	3.82	2.04	3.78	2.02
Childhood stressors					0.18	0.42	0.19	0.43
EA stressors					0.49	0.65	0.50	0.67
Later life								
Resilience					21.7	4.2	22.1	4.4
Health conditions					3.13	1.83	3.15	1.79
Medications					4.14	3.06	3.87	3.02
HADS					7.31	4.27	6.90	4.37
WEMWBS					54.7	7.0	55.2	6.4
Loneliness					0.76	0.91	0.61	0.91
NART							35.1	8.0
RSPM							33.7	7.4

*SMS1947* Scottish mental survey of 1947, *MHT* Moray House test; *SB IQ* Stanford Binet intelligence quotient, *EA* early-adulthood, *HADS* hospital anxiety and depression scale, *WEMWBS* Warwick-Edinburgh mental well-being scale, *NART* national adult reading test, *RSPM* Raven’s standard progressive matrices. Participants who reported a diagnosis of dementia were excluded from the two later subsamples

### Measures

#### Childhood IQ

Participants in the SMS1947, including 1112 of the 6-Day Sample, completed the Moray House Test No. 12 [30]. We derived an IQ-type score from this test by adjusting for age and standardising with a mean of 100 and SD of 15 (MHT IQ). Shortly after the SMS1947, members of the 6-day sample also completed Form L of Terman and Merrill’s 1937 revision of the Stanford–Binet Test [26, 31], providing a second measure of childhood intelligence (SB IQ).

#### Childhood illness

Participants underwent a medical examination in 1950, and notes on physical health were taken during home visits in 1951, 1953 and 1954 [27]. The number of serious illnesses recorded at these times was used as a measure of childhood illness.

#### Dependability

At around the same time as the medical examination, the 6-day sample members’ teachers rated them on six personality characteristics, each on a five-point scale. These characteristics—‘self-confidence’, ‘perseverance’, ‘stability of moods’, ‘conscientiousness’, ‘originality’ and ‘desire to excel’—were selected from a longer list of traits devised by Terman [32]. Deary et al. [24] previously subjected the 6-Day Sample’s adolescent characteristic ratings to principal component analysis (PCA), identifying a component they denoted ‘dependability’, on which perseverance, stability of moods and conscientiousness loaded heavily. We also used the first unrotated component underlying the six characteristic ratings as a measure of dependability.

#### Social class

We derived childhood social class from participants’ fathers’ occupations, recorded in 1947 [26], and coded according to the UK’s 1951 classification of occupations [33, 34], giving a score from 1 to 5.

#### Family environment

Family size and birth order were recorded shortly after the SMS1947 [26]. The numbers of rooms and residents in each participant’s household were also recorded annually from 1950 to 1954 [27]. Home occupancy rate was calculated for each year, dividing number of residents by number of rooms, and then averaged across years.

#### Childhood stressors

Information on parents’ deaths, divorces and separations was recorded systematically through the questionnaire booklet completed in 2013. Any occurring before the age of 18 were counted as childhood stressors. The sum of school moves, recorded in 1947 [26] and 1950 [27], and home moves, recorded annually up until 1963 [23], was used separately as a measure of early-life mobility. Home and school moves have previously been linked to poorer health [35] and academic performance [36], possibly due to associated stress.

#### Early-adulthood stressors

Participants’ occupations were recorded annually up until age 27. Divorces were recorded retrospectively when General Register House records were checked in 1969. Any periods of unemployment, a high number of changes of occupation before age 27, divorce before age 30 and death of a parent between 18 and 30 were counted as early adulthood stressors. Childhood stressors and early-adulthood stressors were also combined as a measure of early-life stressors.

#### Resilience

The Brief Resilience Scale (BRS) [4] was incorporated into the questionnaire booklet. It comprises six statements, three positively worded, e.g., ‘I tend to bounce back quickly after hard times’, and three negatively worded, e.g., ‘I have a hard time making through stressful events’. Responses to each are provided on a five-point scale from ‘strongly disagree’ to ‘strongly agree’, converted to a score between 1 and 5, and summed to produce a total score out of a possible 30.

#### Older-age health

The questionnaire booklet included a series of questions on health conditions and prescribed medications, and one asking participants to rate their own health on a five-point scale from ‘Excellent’ to ‘Poor’. During the telephone interview, participants also completed Townsend’s Functional Ability Scale [37], a nine-item assessment of the impact of health on daily living. Through PCA, we derived a strong single component explaining 57.3 % of the variance in number of health conditions (component loading 0.77), number of medications (0.80), self-rated health (0.81) and functional ability (0.65), which we used as a measure of older-age health.

#### Older-age wellbeing

The questionnaire booklet also included the 14-item Hospital Anxiety and Depression Scale (HADS) [38] and the 14-item Warwick-Edinburgh Mental Well-Being Scale (WEMWBS) [39]. Total scores were used as measures of poor mental health and subjective wellbeing, respectively. Additionally, a single-item measure of loneliness asked participants to indicate, on a five-point scale from ‘Never’ to ‘Most of the time’, how often they felt lonely.

#### Older-age cognitive ability

During the telephone interview, participants completed the National Adult Reading Test (NART) [40], a 50-item vocabulary test, and Raven’s standard progressive matrices (RSPM) [41], a 60-item test of non-verbal reasoning. Test materials were sent to participants in a sealed envelope, which they were instructed to keep closed until the interview [25]. The residual of RSPM score over SB IQ was also used as a measure of lifelong cognitive change.

#### Analysis

Data were analysed in Matlab (2013a; MathWorks, Natick, MA). Three participants who reported a diagnosis of dementia were first excluded, and outlying data points were capped at three SDs from the mean. We used unpaired *t*-tests to check for gender differences in resilience and later-life outcomes, then assessed relations between early-life factors and resilience in older age by computing Pearson’s and Spearman’s correlation coefficients. Early-life variables predicting older-age resilience with a *p* value below 0.1 were then entered together into a generalised linear model of resilience. A separate model was used for combined early-life stressors. We assessed older-age correlates of resilience in the same way, first testing bivariate associations, then entering all those with a *p* value of less than 0.1 into a multivariate model of resilience. We then assessed the bivariate associations between early-life predictors and later-life correlates of resilience, tested whether each significant association was mediated by resilience using multiple regression (as per Baron and Kenny’s [42] method), and assessed the strength of any mediating effects using a Sobel test. Results with a *p* value below 0.05 are reported as significant, but are also considered in terms of the increased chance of making a Type 1 error due to multiple comparisons.

## Results

Descriptive statistics are presented in Table 1. More females than males were involved in the follow-up study, but there were no significant gender differences in resilience (*t* = 1.23, *p* = 0.210) or any other later-life outcome (all *p* > 0.115). Subsequent analyses were therefore performed for males and females together.

We first assessed the longitudinal relationships between early-life factors and resilience in older age (Table 2). Measures of childhood IQ showed only weak positive correlations with later resilience, which were not significant. Resilience did, however, show a stronger and significant negative correlation with teachers’ ratings of adolescent dependability (*r* = −0.18, *p* = 0.021). Childhood illness was significantly negatively associated with older-age resilience (*ρ* = −0.16, *p* = 0.034). Childhood social class did not predict later-life resilience, nor did family size. Birth order showed a weak negative correlation, as did home and school moves throughout early life, while home occupancy rate showed a weak positive association—but none of these effects achieved significance. Childhood stressors significantly predicted higher resilience in older age (*ρ* = 0.16, *p* = 0.034), and early-adulthood stressors also showed a modest positive association close to achieving significance (*ρ* = 0.14, *p* = 0.062). Combined early-life stressors showed a moderate positive correlation with later-life resilience (*ρ* = 0.23, *p* = 0.003).

**Table 2:** Early-life predictors of resilience in older age

Predictor	Bivariate		Multivariate		
	*r*	*p*	*β*	SE	*p*
SB IQ	0.12	0.130			
MHT IQ	0.10	0.218			
Dependability	**−0.18**	**0.021**	−0.13	0.076	0.085
Childhood illnesses	**−0.16**	**0.034**	**−0.18**	**0.074**	**0.018**
Social class	0.05	0.517			
Family size	−0.04	0.625			
Birth order	−0.10	0.186			
Home occupancy	0.11	0.149			
Home/school moves	−0.11	0.174			
Childhood stressors	**0.16**	**0.034**	**0.19**	**0.075**	**0.012**
Early-adulthood stressors	0.14	0.062	0.15	0.077	0.052
Early-life stressors	**0.23**	**0.003**	**0.21**	**0.076**	**0.008**

*SB* Stanford–Binet, *IQ* intelligence quotient, *MHT* Moray House test. Multivariate results were derived from a generalised linear model including all predictors showing a bivariate association of *p* < 0.1. The combined total of early-life stressors was modelled separately from the contributing totals of childhood and early-adulthood stressors. Significant results at *p* < 0.05 (without correcting for multiple comparisons) are highlighted in bold

Overall, five of the assessed early-life variables were significant (or near-significant) predictors of resilience: adolescent dependability, childhood illness, childhood stressors, early-adulthood stressors and combined early-life stressors. These variables were entered together into a generalised linear model of resilience. After controlling childhood illnesses and early-life stressors, adolescent dependability still had a weak negative effect on resilience in older age, but this was no longer significant. However, controlling dependability and early-life stressors, childhood illness remained significantly negatively associated with older-age resilience (*β* = −0.18, *p* = 0.018). Controlling dependability, illnesses and each other, number of childhood stressors remained a significant predictor of higher older-age resilience (*β* = 0.19, *p* = 0.012), and early-adulthood stressors still showed a positive association close to achieving significance. After controlling dependability and illness in a separate model, the combined total of early-life stressors also remained a significant predictor of higher resilience in later life (*β* = 0.21, *p* = 0.008).

To investigate the importance of resilience in later life, we also assessed relations between resilience and other older-age factors. As shown in Table 3, resilience was positively correlated with health in older age (*r* = 0.25, *p* = 0.005), and showed a relatively strong negative correlation with scores on the HADS (*r* = −0.46, *p* < 0.001; Table 3). Resilience was also associated with higher subjective wellbeing, as measured by the WEMWBS (*r* = 0.41, *p* < 0.001), and negatively correlated with loneliness (*r* = −0.23, *p* = 0.003). Older-age cognitive abilities were weakly positively associated with resilience, but not significantly, and cognitive change over 66 years showed no association. As before, we entered significant correlates of resilience—health, mental health problems, wellbeing and loneliness—together into a multivariate model. After controlling other older-age correlates of resilience, HADS score still showed a moderate negative association with resilience (*β* = −0.32, *p* = 0.004). Wellbeing and loneliness still showed weak but not significant associations, while older-age health no longer showed any association.

**Table 3:** Correlates of resilience in older age

Correlate	Bivariate		Multivariate		
	*r*	*p*	*β*	SE	*p*
Older-age health	**0.25**	**0.005**	−0.01	0.097	0.911
HADS	**−0.46**	**<0.001**	**−0.32**	**0.109**	**0.004**
WEMWBS	**0.41**	**<0.001**	0.14	0.102	0.180
Loneliness	**−0.23**	**0.003**	−0.10	0.089	0.249
NART	0.13	0.138			
RSPM	0.07	0.436			
Cognitive change	0.00	0.991			

*HADS* Hospital anxiety and depression scale, *WEMWBS* Warwick-Edinburgh mental well-being scale, *NART* national adult reading test, *RSPM* Raven’s standard progressive matrices. Multivariate results were derived from a generalised linear model including all correlates showing a bivariate association of *p* < 0.1. Significant results at *p* < 0.05 (without correcting for multiple comparisons) are highlighted in bold

Finally, we explored associations between early-life predictors and later-life correlates of resilience. For significant associations, we tested whether they were mediated by resilience (Table 4). Childhood illnesses predicted poorer health in older age (*ρ* = −0.20, *p* = 0.026), and positively correlated with HADS score (*ρ* = 0.19, *p* = 0.013). Multivariate analyses indicated that, for each outcome, both childhood illness and resilience had significant independent associations, suggesting resilience partly mediated the effects of childhood illness on older-age health and mental health problems. This mediating effect did not achieve significance for health (*z* = 1.75, *p* = 0.080), but did for mental health problems (*z* = 2.09, *p* = 0.036). Early-adulthood (*ρ* = −0.24, *p* = 0.002) and early-life (*ρ* = −0.21, *p* = 0.005) stressors showed significant negative correlations with loneliness in older age. Modelled alongside resilience, each still showed a significant association, while, in each case, resilience did as well, again suggesting partial mediation. The mediating effect was significant for combined early-life stressors (*z* = 2.23, *p* = 0.026), but not for early-adulthood stressors separately.

**Table 4:** Associations between early-life predictors and older-age correlates of resilience, and their mediation by resilience

Predictor	Outcome	Bivariate		Indirect		
		*r*	*p*	*β*	*z*	*p*
Dependability	Older-age health	0.06	0.533			
	HADS	−0.04	0.574			
	WEMWBS	−0.04	0.575			
	Loneliness	0.12	0.116			
Childhood illnesses	Older-age health	**−0.20**	**0.026**	−0.04	−1.75	0.080
	HADS	**0.19**	**0.013**	**0.08**	**2.09**	**0.036**
	WEMWBS	−0.12	0.130			
	Loneliness	−0.03	0.671			
Childhood stressors	Older-age health	0.13	0.143			
	HADS	−0.03	0.678			
	WEMWBS	0.14	0.074			
	Loneliness	−0.00	0.953			
EA stressors	Older-age health	−0.07	0.442			
	HADS	−0.02	0.756			
	WEMWBS	0.04	0.649			
	Loneliness	**−0.24**	**0.002**	−0.03	1.63	0.103
Early-life stressors	Older-age health	0.01	0.874			
	HADS	−0.04	0.594			
	WEMWBS	0.11	0.138			
	Loneliness	**−0.21**	**0.005**	**−0.05**	**2.23**	**0.026**

*EA* Early-adulthood, *HADS* hospital anxiety and depression scale, *WEMWBS* Warwick-Edinburgh mental well-being scale. The mediating effects of resilience were assessed using the Sobel test for all bivariate associations of *p* < 0.1. Significant results at *p* < 0.05 (without correcting for multiple comparisons) are highlighted in bold

## Discussion

In this study, we explored the contribution of a range of early-life factors, some assessed as early as age 11 years, to resilience in later life, assessed at 77 years. Childhood intelligence, social class, family environment, and home and school moves, showed no significant association. However, higher adolescent dependability and a higher number of childhood illnesses significantly predicted lower older-age resilience, while a higher number of early-life stressors significantly predicted higher resilience. Bivariate results were not corrected for multiple comparisons, but when predictors were entered together into a multivariate model of resilience, independent effects of childhood illness, childhood stress and total early-life stress remained significant. Resilience in turn was related to health, mental health, wellbeing and loneliness in older age. It was also a significant mediator of the relationships between childhood illness and older-age mental health, and early-life stressors and older-age loneliness.

The significant negative correlation between adolescent dependability and older-age resilience demonstrated an association between personality and resilience over more than six decades. This is consistent with previous work demonstrating a contemporaneous relationship [9–12], but is the first evidence of an association over such a long interval. In another recent study of data from the 6-day sample [43], adolescent dependability showed no significant correlation with its corresponding ratings or other personality measures in older age. This suggests the longitudinal relationship between personality and resilience in the present study was not simply due to the lifelong stability of personality and a contemporaneous relationship between the two in older age. Instead, perhaps more complex intermediary effects throughout life are responsible for this result. However, this correlation was negative, i.e., higher dependability predicted lower resilience. This seems unexpected, because previous work has shown a positive contemporaneous relationship between comparable personality traits and resilience [9–12]. This also indicates that the longitudinal association between resilience and dependability might be attributable to complex mechanisms operating throughout life. The relationship between resilience and other personality traits may be different at various stages of life, an important consideration for studying related constructs in participants of other ages.

Childhood illness also showed a negative relationship with later-life resilience, even after controlling other factors. This relationship, however, might be expected to be negative. Previous studies have observed a negative relationship between illness and psychological resilience [4–8], although this is normally interpreted as a sign of resilience protecting against the development of illnesses. If those of our participants higher in resilience in older age were already more resilient in childhood, this could have protected against childhood illness and then been retained throughout life into older age. Alternatively, childhood illness could have had an impact on physical resilience, by increasing expectations of succumbing to illness again in the future, and psychological resilience may also have been impaired if this susceptibility was perceived as extending to other stressors.

We also found that stressors throughout early life predicted higher resilience in later life. This may seem counterintuitive, considering the well-established relationships between early-life trauma and later behavioural and emotional problems, and psychiatric disorders [20, 21, 44–47]. However, stress does not lead to such problems in all cases [14],—perhaps not even in most cases [48]. Furthermore, it seems plausible that learning to adapt to stress effectively would require experience of stress [16], and previous results such as those of Shapero et al. [19], suggest moderate childhood stress protects against the later development of psychiatric problems. This has also been demonstrated through controlled animal experiments, allowing direct manipulation of stressors [18, 49]. Importantly, these prior studies of stress inoculation assessed brief or moderate stressors, rather than chronic stress. However, Cicchetti and Rogosch [50] found that even a stressor as severe and prolonged as childhood physical abuse can lead to higher resilience. Our findings support the stress inoculation hypothesis, and, although none of our stressors were as severe as abuse, some were more severe or more prolonged than the stressors used in previous stress inoculation research, and still had a positive effect on later resilience.

Middlebrooks and Audage [22] differentiate between ‘toxic stress’ in childhood, usually relating to long-term neglect or abuse; ‘tolerable stress’, such as the death of a loved one or family disruption; and ‘positive stress’, relating to minor everyday stressors. They suggest that positive stress is helpful in developing coping mechanisms for dealing with stress in later life, and that, with the right pre-existing coping mechanisms and support from others, tolerable stress can act as positive stress too. Although some of the early-life stressors counted in this study seem severe, none would be categorised by Middlebrooks and Audage as toxic, so this model suggests all could have benefited resilience. We argue that participants in the present study who were exposed to higher levels of tolerable stress as children and young adults, through recovering from the associated trauma, developed greater resilience. Some members of the original 6-day sample may also have experienced higher levels of stress in childhood and early adulthood but without developing higher resilience. However, their lower resilience (and, by association, lower physical and mental wellbeing) could have made them less likely to participate in the follow-up study. Nonetheless, our results demonstrate that stress can lead to greater resilience, even if it does not in every case.

In light of this, we can now briefly revisit the negative relationship we observed between adolescent dependability and later-life resilience. Although one might expect a positive relationship between dependability and resilience, a negative relationship between dependability and early-life stressors might also be assumed; early-life stress has previously been associated with higher negative traits, such as neuroticism, and lower positive traits, such as conscientiousness [51, 52]. If childhood stressors were associated with lower dependability in adolescence, as expected, and then also predicted higher resilience in later-life, as we observed, then a negative longitudinal relationship between dependability and resilience would be expected as well.

Resilience has previously been associated with physical and mental health problems [4–8]. Consistent with such findings, we observed moderate correlations between resilience and health, mental health and wellbeing in older age. Although resilience and other older-age outcomes were assessed at the same time, which means that the direction of causation cannot be inferred with any certainty, these results may demonstrate the importance of resilience to later-life outcomes, the usefulness of resilience scales as prognostic tools, and the potential of resilience as a target for therapeutic intervention. They also suggest that the resilience measure used was reasonably accurate. Although only mental health showed a significant independent association when later-life variables were included together in a multivariate model of resilience, this may simply reflect the degree of interrelation among mental health, physical health, wellbeing and loneliness [53–57]; the significant bivariate associations still represent valid results. Additionally, resilience significantly mediated the relationships between early-life stress and loneliness in older-age, and between childhood illness and older-age mental health. These results provide stronger evidence to support targeting resilience in therapeutic interventions.

Although the original 6-day sample was almost perfectly representative of the Scottish 1936 birth cohort, not all of the original participants were available and volunteered to take part in the follow-up study. Those who did differed significantly from the whole sample in terms of childhood factors such as IQ and dependability, as described by Johnson, Brett, Calvin and Deary [29]. A principal reason for original participants being excluded from the follow-up study was mortality—over 400 of the original sample died prior to the beginning of the follow-up study—which would of course affect any study of older people similarly. However, in this study, the main variables of interest were all related to mortality. Dependability [24] and resilience [58] both predict longevity, so original 6-day sample members low in both would be more likely to have died before the follow-up study. Participants low in dependability may have survived through to older-age due to higher resilience, and vice versa, which could explain the negative correlation we observed between the two. This provides further reason to also consider personality traits in future studies of resilience.

A related limitation was the size of the sample, which may not have provided enough power to identify more subtle predictors of later resilience. Also, the original 6-day sample was first studied for completely different reasons, almost seven decades ago, which meant some of the early-life measures were not perfect. We were able to create a measure of early-life stress from the data collected, but many more early-life stressors were not systematically recorded, and personality was not assessed within the context of what might today be called a standard model. It would be interesting to see how the availability of more established measures or more systematic assessment of these constructs would have affected our results. Future investigation of the relationship between early-life stress and resilience in particular should also consider subjective experience of stress, or physiological measures of stress response (e.g., cortisol levels), as various potential stressors will affect individuals differently. Likely generational differences in the impact of the stressors that we studied should also be considered; for example, parental separation is now more common and usually less traumatic than it would have been six decades ago. Finally, our measure of resilience may represent another limitation, as it consisted of only six items. However, the BRS has been validated as a measure of resilience previously [4], and it did relate to contemporaneous health and wellbeing measures as expected in this study.

Our main finding, that early-life stress predicts resilience in later-life, provides support for the stress inoculation hypothesis. This should, however, be considered within the context of the sample and measures we used. In many people, early-life trauma can have devastating long-term consequences, such as psychiatric disorders—but these people are less likely to have participated in our follow-up study at age 77. Among those who did take part, resilience may still have been lower among those exposed to more severe stressors that were not recorded. Our results suggest that early-life stress can lead to the development of higher resilience, rather than that it consistently does. Further research is required to identify factors determining whether or not people who experience early-life stress subsequently become more resilient, and other ways in which resilience might be enhanced. This is of particular importance, considering our other notable finding, that resilience mediates the effects of early-life factors on later-life health and wellbeing outcomes. Better understanding of the development of resilience throughout life may be an important stepping stone for research focused on improving health and wellbeing in older-age.
